# Molecular red cell genotyping of rare blood donors in South Africa to enhance rare donor-patient blood matching

**DOI:** 10.4102/ajlm.v10i1.1400

**Published:** 2021-09-27

**Authors:** Lavendri Govender, Rosaley D. Prakashchandra, Pavitra Pillay, Ute Jentsch

**Affiliations:** 1 Department of Biomedical and Clinical Technology, Faculty of Health Sciences, Durban University of Technology, Durban, South Africa; 2 Molecular Research and Development Department, Specialised Laboratory Services, South African National Blood Service, Durban, South Africa; 3 Medical Department, Specialised Laboratory Services, South African National Blood Service, Durban, South Africa

**Keywords:** red cell genotyping, genetic variations, rare blood types, donor-patient blood matching

## Abstract

**Background:**

Molecular red cell genotyping is devoid of serology limitations such as the scarcity of rare antisera and the possibility of inconclusive results due to biological interferences. Blood incompatibility can result in immune transfusion reactions such as haemolytic transfusion reactions or haemolytic disease of the foetus and newborn.

**Objective:**

The study aimed to use molecular red cell genotyping to identify rare blood group donors among South African blood donors.

**Methods:**

Red cell genotyping data were extracted retrospectively from the BIDS XT genotyping software in the Immunohaematology Reference Laboratory from January 2015 to August 2016. The ID CORE XT genotyping assay was used to identify the single nucleotide polymorphisms of 10 blood groups system alleles in 150 donors. Associations between the resultant genotypes and predicted phenotypes, ABO group, RhD type, race group and gender were studied.

**Results:**

Significant red cell genetic variability was noted among the numerous South African donor genotypes identified in this study. Genotyping further confirmed the presence of at least one of the 16 rare genotypes in 50 donors. Group O Black donors were associated with two rare blood types, while several other rare blood types were found only in White donors, supporting an association between ABO/Rh subtype, race group and rare blood types.

**Conclusion:**

Targeted screening of donors for antigen-negative rare blood units for patients should be done to reduce the risk of haemolytic transfusion reactions and haemolytic disease of the foetus and newborn.

## Introduction

The antigens found on the surface of red blood cells determine the blood type of an individual. Blood grouping is essential for donor-patient blood transfusion match.^1^ Red cell antigen mismatch between donor and patient can result in alloimmunisation, which is the formation of red cell antibodies in the patient following transfusion with red cells that the patient lacks. Clinically, alloimmunisation can result in mild to fatal haemolytic transfusion reactions.^2^ In neonates, alloimmunisation may cause mild to fatal haemolytic disease of the foetus or newborn.^3,4^

The current serological phenotyping method of red cell antibody detection used in South Africa is simple and inexpensive. However, inconclusive serology results may result due to the interference from donor red blood cells in chronically transfused patients, a rare blood type causing false-positive results or autoantibodies in patients with autoimmune diseases.^5^ Also, sourcing blood for patients with rare blood types is challenging due to the scarcity of commercial rare antisera for serological testing.^6^ The term rare blood type describes the absence of an antigen that is found normally in 99% of the country’s population and is described as: ‘negative for high-frequency-antigens (HFA)’ or the presence of antigens found in only 1% of the population termed ‘positive for low-frequency-antigens’ or the presence of an unusual or rare Rh subtype in a very small percentage of the population (1:1000, 1:10 000).^7^ Hence, rare commercial antisera are often not readily available and are expensive. Therefore, they are not cost-effective for large-scale blood type screening.^6^

Molecular genotyping overcomes the limitations of serology. The mapping of the human genome provided knowledge on the molecular backgrounds and polymorphisms of the blood groups that enabled the development of DNA-based assays for red cell genotyping.^8^ Genotyping utilises patients’ DNA information to infer the red cell phenotype. Genotyping assays have been widely used in transfusion medicine for over 20 years.^8^ This technology only became an affordable service in South Africa in 2015 and has to date not translated into the genetic identification of rare and unique red cell genotypes in South Africa or the wider African context. Smaller studies in some African countries have been completed; however, these were limited to group-specific antigen coverage. Moreover, the sample numbers were too low across the regions to be representative of the African diaspora and its multiple ethnic groups.^9^ Therefore, this study aimed to provide the first comprehensive red cell genotyping of rare blood donors referred to the South African National Blood Service, Immunohaematology Reference Laboratory (IRL).

## Methods

### Ethical considerations

Full approval (ethical clearance number IREC 011/17) was granted by the Durban University of Technology to complete the study as part of a master’s thesis. Ethical approval was also granted by the South African National Blood Service Human Research Ethics Committee (certificate clearance number 2016/06). Initial donor consent was provided by donors when completing the blood donor questionnaire at the time of blood donation. However, there were no human participants in this study as it was a retrospective review of red cell genotyping data only. All genotyping results were anonymised from donor details and blinded using designated study numbers only accessible to the researcher.

### Study participants

The South African National Blood Service provides a vein-to-vein blood service in eight of the nine South African provinces and the majority of the blood donations are successfully cross-matched for transfusion to patients. Where routine cross-matching cannot be completed due to inconclusive serology results, the blood donation is referred to the national Immunohaematology Reference Laboratory. This observational study retrospectively reviewed red cell genotyping data of 150 South African National Blood Service donors whose blood samples were referred to the Immunohaematology Reference Laboratory between January 2015 and August 2016.

The study population of 150 donors comprised 58 conveniently selected serology-determined rare blood type donors and 92 randomly selected Group O, RhD+ donors (Table 1). The selection bias for Group O+ donors was because the South African National Blood Service donor recruitment strategy focuses on sourcing mainly Group O+ blood since it is a common blood type in South Africa and can be transfused to A+, B+, AB+ and O+ patients.

**TABLE 1:** Percentage occurrence of the ID CORE XT predicted phenotypes and genotypes for 150 South African blood donors genotyped at the South African National Blood Service Immunohaematology Reference Laboratory from January 2015 to August 2016.

Blood groups†	ID Core XT		Occurrence (*n* = 150)		Race			
	Predicted phenotype	Genotype	*n*	%	White (*n* = 61)	Black (*n* = 59)	Indian (*n* = 19)	Mixed race (*n* = 11)
Rh-negative	ce/ce (rr)	RHCE*ce, RHCE*ce	11	7.3	10	-	1	-
	Ce/ce (r’r)	RHCE*Ce, RHCE*ce (+2 other genotypes)	8	5.4	1	4	-	3
	Ce/Ce(r’r’)	RHCE*Ce, RHCE*Ce	1	0.7	1	-	-	-
	Ce/cE(r’r”)	RHCE*Ce, RHCE*cE	4	2.7	1	-	3	-
	ce/cE (r”r)	RHCE*cE, RHCE*ce	1	0.7	1	-	-	-
Rh-positive	ce/ce (R_o_)	RHCE*ceAR, RHCE*ceAR (+9 other genotypes)	57	38.0	10	42	1	4
	cE/ce (R_2_r)	RHCE*cE, RHCE*ce (+2 other genotypes)	18	12.0	9	7	1	1
	Ce/Ce(R_1_R_1_)	RHCE*Ce, RHCE*Ce	19	12.5	7	-	10	2
	cE/cE(R_2_R_2_)	RHCE*cE, RHCE*cE	11	7.3	6	4	1	-
	Ce/ce (R_1_r)	RHCE*Ce, RHCE*ce	12	8.0	8	2	2	-
	Ce/cE (R_1_R_2_)	RHCE*Ce, RHCE*cE	6	4.0	6	-	-	-
	Ce/CeCw(R_1_R_1_Cw)	RHCE*Ce, RHCE*CeCW	1	0.7	1	-	-	-
	CE/Ce(R_z_R_1_)	RHCE*CE, RHCE*Ce	1	0.7	-	-	-	1
Kell	k+ Kpb+ Jsb+	KEL*k_KPB_JSB, KEL*k_KPB_JSB	126	84	51	48	17	10
	k– Kpb+ Jsb+	KEL*K_KPB_JSB,KEL*K_KPB_JSB	6	4.0	5	-	1	-
	k+ Kpb+ Jsb–	KEL*k_KPB_JSA, KEL*k_KPB_JSA	6	4.0	-	5	1	-
	k+ Kpb+ Jsa+ Jsb+	KEL*k_KPB_JSB, KEL*k_KPB_JSA	6	4.0	-	5	-	1
	K+ k+ Kpb+ Jsb+	KEL*K_KPB_JSB,KEL*k_KPB_JSB	5	3.3	5	-	-	-
	k+^c^ Kpa+ Jsb+§	KEL*k_KPA_JSB, KEL*k_KPA_JSB	1	0.7	1	-	-	-
Kidd	Jka+, Jka+	JK*A, JK*A	75	50.0	18	43	10	4
	Jka+, Jkb+	JK*A, JK*B	48	32.0	24	14	7	3
	Jkb+, Jkb+	JK*B, JK*B	27	18.0	19	2	2	4
Duffy	Fya–, Fyb–‡	FY*B_GATA, FY*B_GATA	47	31.3	5	39	1	2
	Fya+ Fyb+	FY*A, FY*B	39	26.0	22	2	10	5
	Fyb+	FY*B, FY*B	27	18.0	20	4	2	1
		FY*B, FY*B_GATA	18	12.0	5	12	-	1
	Fya+ Fyb–	FY*A, FY*A	14	9.3	8	-	6	-
		FY*A, FY*B_GATA(1)	4	2.7	-	2	-	2
	Fya+ Fyb§	FY*A, FY*B[265T]_FY*X	1	0.7	1	-	-	-
MNS – MN	M+, N+	GYPA*M, GYPA*N	73	48.7	30	28	11	4
	M+, M+	GYPA*M, GYPA*M	55	36.7	22	21	7	5
	N+, N+	GYPA*N. GYPA*N	22	14.7	10	9	1	2
MNS – S-s-U	S-s+	GYPB*s, GYPB*s	74	49.3	30	29	13	2
		GYPB*s, GYPB*S_null(IVS5+5t)	1	0.7	-	1	-	-
		GYPB*s, GYPB*S_null(230T)	1	0.7	-	1	-	-
	S+ s+	GYPB*S, GYPB*s	36	24.0	15	17	2	2
	S+, S+	GYPB*S, GYPB*S	27	18.0	6	5	4	12
		GYPB*S, GYPB*S_null(IVS5+5t)	2	1.3	-	-	-	2
	S-s-U-variant	GYPB*S_null(IVS5+5t), GYPB*S_null(IVS5+5t)	5	3.3	1	3	-	1
	S-s-U-variant	GYPB*S_null(230T), GYPB*S_null(IVS5+5t)	1	0.7	-	1	-	-
	S-s-U-	GYPB*deletion, GYPB*deletion	3	2.0	1	-	-	2
Diego	Dia–, Dib+	DI*B, DI*B	150	100.0	61	59	19	11
Dombrock	Dob+, Dob+	DO*B, DO*B	67	44.7	25	31	8	3
		DO*B, DO*B_HY	6	4.0	-	6	-	-
		DO*B_HY, DO*B_HY	1	0.7	1	-	-	-
	Doa+, Dob+	DO*A, DO*B	46	30.7	22	16	4	4
		DO*B, DO*A_JO	4	2.7	-	4	-	-
		DO*A, DO*B_HY	1	0.7	-	1	-	-
	Doa+, Doa+	DO*A, DO*A	23	15.3	12	-	7	4
		DO*A, DO*A_JO	1	0.7	-	1	-	-
		DO*A_JO, DO*A_JO	1	0.7	1	-	-	-
**Colton**	**Coa+, Coa+**	CO * A, CO * A	**146**	**97.3**	**57**	**59**	**19**	**11**
	Coa+, Cob+	CO*A, CO*B	4	2.7	4	-	-	-
Cartwright	Yta+, Yta+	YT*A, YT*A	142	94.7	58	59	14	11
	Yta+, Ytb+	YT*A, YT*B	7	4.7	3	0	4	0
	Ytb+, Ytb+	YT*B, YT*B	1	0.7	0	0	1	0
Lutheran	Lub+, Lub+	LU*B, LU*B	140	93.3	57	57	19	7
	Lua+, Lub+	LU*A, LU*B	9	6.0	3	2	0	4
	Lua+, Lua+	LU*A, LU*A	1	0.7	0	1	0	0

Note: The ID CORE XT only covers the Rh group RHCE gene coding for the C, c, E, e, V, VS antigens; thus, the RhD positive and negative result was based on the donors’ serology Rh result. The MNS blood group main antigens are noted separately as MN and S-s-U as this is how the ID CORE XT identifies these antigens.

+, denotes positive or presence of the antigen; –, denotes negative or absence of the antigen;

† , 10 blood groups are listed as covered by the ID CORE XT assay;

‡ , Fya-b– phenotype is not reflective of the rare Fya-b- phenotype but is due to the GATA mutation detected by the ID CORE XT assay;

§ , indicates partial or variant allele expression as per the package insert of the ID CORE XT assay.

The self-proclaimed race groups of the donors as stated on the blood donation donor questionnaire were collated to establish whether there is an association between red cell genotypes, rare blood types and race groups. Donors indicated their self-proclaimed race groups as either Black, mixed race, Indian or White.^10^ These racial groups were introduced by apartheid and remain a description of the South African society with Black South Africans being the native Black African population, mixed race South Africans describing a person of mixed European (‘White’) and African (‘Black’) or Asian ancestry, as officially defined by the South African government from 1950 to 1991, Indian South Africans are South Africans who descend from migrants who arrived from British-ruled India during the late 1800s and early 1900s and White South Africans are South Africans of European descent, as well as from certain parts of West Asia. Since the samples of donors were collected nationally within South Africa, all race groups have representation in the study although not of equal distribution.

### DNA extraction

DNA was extracted from blood collected in ethylene-diamine-tetraacetic-acid anticoagulated blood tubes using the Maxwell AS2000 (Promega, Madison, Wisconsin, United States) instrument and the Promega Maxwell standard elution volume assay. DNA was quantified using the Nanodrop 2000 (ThermoFisher Scientific, Waltham, Massachusetts, United States) then diluted to obtain a standard DNA concentration of 20ng/µL with a purity falling between 1.7 and 1.9 as required for completion of the ID CORE XT assay (Progenika, Derio, Spain).

### ID CORE XT assay

To maximise resources, the Immunohaematology Reference Laboratory utilised the ID CORE XT assay kit (Progenika/Grifols, San Antonio, Texas, United States). The ID CORE XT kit covers red cell antigens or alleles of 10 blood grouping systems: RHCE, Kell, Kidd, Duffy, MNS, Diego, Dombrock, Colton, Cartwright and Lutheran, totalling 37 red cell antigens in a single test. The ID CORE XT kit was assayed in a Luminex 200IS analyser (Luminex Corporation, Austin, Texas, United States) to detect beads coated with specific single nucleotide polymorphisms. The BIDSXT assay was used to exclude all invalid red cell genotypes that did not yield a bead count of greater than 30 or a median fluorescent intensity of 1000.

### Data analysis

The predicted phenotypes and genotypes were exported from the BIDS XT (Progenika/Grifols, San Antonio, Texas, United States) software to a PowerBI software programme. Simultaneously, demographic details of gender, race, ABO group and RhD types were exported from the organisation’s information system to the business intelligence information technology database to study associations between these characteristics.

### Database collation

The occurrence of the genotypes and predicted phenotypes were analysed from the most to the least occurring among the White, Black, Indian and mixed South African race groups. Rare blood types are either negative for HFA, positive for low-frequency-antigens or unusual, rare Rh subtypes that occur in less than 1% in a population. However, due to the small sample size of the study and the 34 rare blood types that were conveniently added to the study, we could not use the less than 1% guideline. Instead, the rare blood types currently listed on the South African Rare Donor file was used to separate the rare blood types – HrB–, HrS–, k–, Jsb–, U–, Kpb–, Yta–, Lub–, Hy–, Joa–, Uvar+, Cw+, r’r”, r”r, r’r’ and R_z_R_1_ – genetically tested in this study.

## Results

**FIGURE 1:**
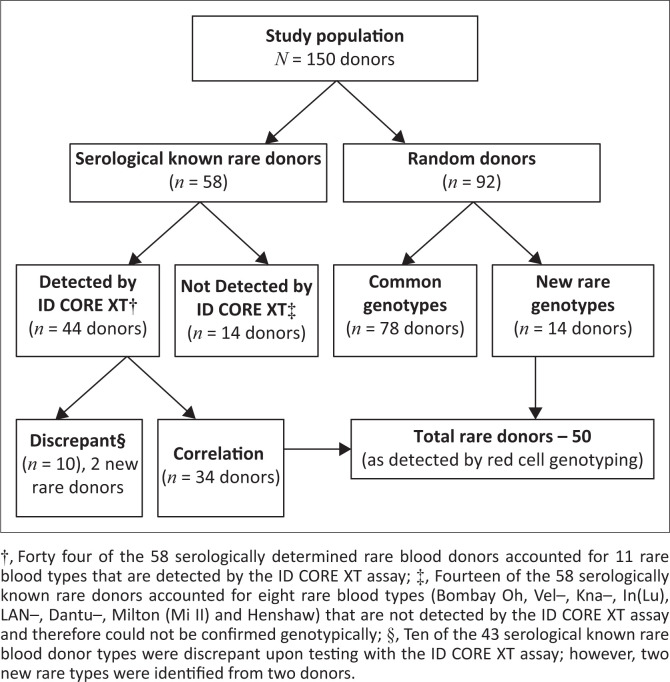
Breakdown of the study population and the resultant total of 50 genetically identified donors based on retrospective red cell genotyping obtained from the South African National Blood Service, Immunohaematology Reference Laboratory over the period January 2015 – August 2016.

Most of the study participants were men (63%, *n* = 95/150) with 80% of the race group distribution comprising 41% (*n* = 61) White donors and 39% (*n* = 59) Black South African donors. The 2015–2016 red cell genotyping data was skewed by a selection bias for Group O+ (71%, *n* = 107) donors which accounted for 33 rare blood types found in 32 donors. In comparison, Group O– donors that made up 13% (*n* = 20) of the study population showed the presence of 15 rare blood types in 14 donors. The remaining 16% (*n* = 23) of the Group A+/− and Group B+/− had four rare blood types in four donors, cumulatively resulting in a total of 50 rare donors with 52 rare blood types at a molecular level. Of the 58 serological known rare donors, 44 donors had the serologically defined rare blood types HrB–, HrS–, k–, U–, U-variant+, Jsb, Kpb–, r̎ r̍, RzR1, Lub– and Yta– that are covered by the ID CORE XT. However, molecular genotype correlated with serotype in only 34 of the 44 donors (Figure 1). Ten of the 44 serology-defined rare blood types were absent upon molecular testing giving a 23% discrepancy rate between serotyping and genotyping. More so, rare blood types previously not detected by serology were found in two of these 10 donors (Figure 1). Fourteen of the 58 serologically known rare donors had one of the eight rare blood types: Bombay Oh, Vel–, Kna–, In(Lu), LAN–, Dantu–, Milton (Mi II) and Henshaw that were not covered in the ID CORE XT assay. Therefore, these could not be confirmed genotypically (Figure 1).

From among the 92 random donors, genetic and molecular screening identified an additional 14 donors with a total of 16 rare blood types. This brings the total number of rare donors confirmed using genotyping to 50, which comprises 33.3% of the study population (Figure 1).

### RhD/RHCE

The most occurring RhD/RHCE phenotype was the Rh-positive Ro (cDe/cDe) phenotype and was found in a majority (72%, *n* = 42/59) of the South African Black donors. Six Black Ro donors were associated with the rare blood genotype RHCE*ceAR, RHCE*ceAR and RHCE*ceAR, RHCE*ce[712G], representing the rare negative HFA, HrS–. In comparison, the White South African donors showed the most genetic variability, having all of the Rh-negative and Rh-positive subtypes. A rare RzR1 (CDE/CDe) phenotype occurred in one mixed race South African donor. The four Black and three mixed race South African donors with the r’r (Cde/cde) phenotype were associated with the rare blood genotype RHCE*ce[733G,1006T], RhD*r’s-RHCE*ce[733G,1006T] that is serologically referred to as the known negative for a HFA Rh:-34 or HrB–. Predominant among the Indian South African donors was the RHCE*Ce, RHCE*Ce, R_1_R_1_ (CDe/CDe) genotype and predicted phenotype.

### Kell

The single nucleotide polymorphisms in ID CORE XT cover the K/k, Kpa/Kpb and Jsa/Jsb antigens from the Kell blood group system (Table 1). The homozygous KEL*k_KPB_JSB, KEL*k_KPB_JSB was the most predominant Kell blood group genotype found across the ethnicities in 84% (*n* = 126) of the study population. The remaining five Kell genotypes were distributed among 16% (*n* = 24) of the race groups with the KEL*k_KPA_JSB, KEL*k_KPA_JSB (serologically referred to as the Kpb-) phenotype being the least common Kell genotype in this study.

### Kidd

The most occurring kidd genotype in the study population was the homozygous JK*A, JK*A Kidd blood type. It was observed in 50% (*n* = 75) of the study population and the majority of the Black and Indian donors. The remaining 50% of the donors had the heterozygous JK*A, JK*B and the homozygous JK*B, JK*B. The three Kidd genotypes were found in almost equal spread among the mixed race donors and the JK*A, JK*B was more common among the White donors.

### Duffy

The predominant occurrence of the GATA mutation indicates the predicted Fya–, Fyb– phenotype as present in 31.3% (*n* = 47) of the study population (Table 1). The FY*B_GATA, FY*B_GATA was predominant among the Black donors while the FY*B, FY*B[265T]_FY*X was observed in one White donor.

### MNS

In the ID CORE XT assay, the GYPA and GYPB genes identifying the MN and S-s-U alleles of the MNS system are identified and reported individually. The relationship between the Ss null alleles resulting in U-antigen variants (U-variant) and the *GYPB* gene deletion resulting in a U-predicted phenotype is evident (Table 1). The rare S-s-U– was found in only the Black donors in this study.

### Diego

No DI*A genotype was detected among study participants; however, the homozygous DI*B, DI*B was detected (Table 1).

### Dombrock, Colton, Cartwright, Lutheran

There was a slightly higher occurrence of the homozygous DO*B, DO*B than the DO*A, DO*B genotype, and there were four Dombrock variants that occurred in less than 1% of the donors. The homozygous CO*A, CO*A, YT*A, YT*A and LU*B, LU*B genotypes occurred in more than 90% of the study population. A small percentage (2.7%, *n* = 4) of White donors had the CO*A, CO*B genotype while the YT*B, YT*B. LU*A, LU*A occurred in less than 1% of donors.

The International Society of Blood Transfusion’s categories of rare blood types include: Negative for HFA, positive for low-frequency-antigens and rare unusual Rh subtypes. There were 10 negative for HFAs detected in 38 donors of which the HrB– and HrS– HFA were most common being found in 12 and 6 donors. The two positive for low-frequency-antigensme and four rare Rh subtypes were found among seven donors. Of the 52 rare blood types listed on Table 2, 1% (*n* = 16) rare blood types were newly identified from 14 donors; two donors had two rare blood types each: HrS– with Jsb– and U– with Dob–. The 1% new rare blood types comprised of 3 HrB–, 3 Jsb–, 2 U-variant (Uvar+), 2 Dob– (DO*A, DO*A_JO), 2 r’r”, 1 HrS–, 1 CeCw, 1 r’r, and 1 U–.

**TABLE 2:** Rare blood types by antigen type and frequency found among 150 donors genotyped between January 2012 and August 2016 at the South African National Blood Service, Immunohaematology Reference Laboratory.

Genotype	Phenotype	*n*	%
**Negative high-frequency-antigens (HFA)** †			
RHCE*ce[733G,1006T], RhD*r’s-RHCE*ce[733G,1006T]‡	Rh:-34,E-HrB–	12	5.3
RHCE*ceAR, RHCE*ce[712G], RHCE*ceAR, RHCE*ceAR¶	Rh:-18 E-HrS–	6	4.0
KEL*K_KPB_JSB, KEL*K_KPB_JSB	k–	6	4.0
KEL*k_KPB_JSA, KEL*k_KPB_JSA	Jsb–	6	4.0
GYPB*deletion, GYPB*deletion	S-s-U–	3	2.0
KEL*k_KPA_JSB, KEL*k_KPA_JSB	Kpb–	1	0.7
YT*B, YT*B	Yta–	1	0.7
LU*A, LU*A	Lub–	1	0.7
DO*B_HY, DO*B_HY	Hy– (Doa–)	1	0.7
DO*A_JO, DO*A_JO	Joa– (Dob–)	1	0.7
**Positive low frequency antigens (LFA)** §			
GYPB*S_null(IVS5+5t), GYPB*S_null(IVS5+5t)	S-s-Uvar+	6	4.0
RHCE*Ce, RHCE*CeCW	Cw+	1	0.7
**Rare Rh subtypes** ††			
RHCE*Ce, RHCE*cE	r’r”	4	2.7
RHCE*ce, RHCE*cE	r”r	1	0.7
RHCE*Ce, RHCE*Ce	r’r’	1	0.7
RHCE*Ce, RHCE*CE	R_z_R_1_	1	0.7

† , Negative for high-frequency antigens – refers to 1% of the country’s population that do not have an antigen or are negative for an antigen that is present in 99% of people;

‡ , RHCE*ce[733G,1006T], RhD*r’s-RHCE*ce[733G,1006T] is one of the ce/ce RHCE genotype found in some Rh-positive (Ro) and R- negative (r’r) donors in the study;

§ , RHCE*ceAR, RHCE*ce[712G], RHCE*ceAR, RHCE*ceAR are two of the ce/ce genotypes found in some of the Rh-positive (Ro) donors;

¶ , Positive for low frequency antigens – refers to 1% of the country’s population that has an antigen or is positive for an antigen that is absent in 99% of people;

†† , Rare Rh subtypes – the ID CORE XT assay does not cover the RhD gene, the serology result for the Rh-positive (shown as *R*) or Rh-negative (shown as r) was used to determine the final phenotype. These subtypes are rare because they are found in less than 1% of the population in a country.

## Discussion

Studying the red cell genetic variation among the four major South African race groups showed that the Rh subtype R_o_ is found in 42 out of 59 Black donors, R_1_R_1_ in 10 out of 19 Indian donors while six different Rh subtypes are spread among the White donors. Also, rare blood types such as the HrB– and HrS– were found more in Black donors while other rare types such as k–, Kpb– were found among White donors suggesting genetical association between Rh subtype, race group and blood type. This knowledge allows for more effective rare donor screening strategies to be implemented to enhance rare donor-patient blood matching in South Africa. In this study, screening of random Group O+ donors identified 14 new rare donors that would have been missed by routine testing. Hence, identifying markers suggesting a rare blood type in donors such as Rh subtype, race and patterns of genetic variations will assist the development of algorithms for rare blood type testing and identification in donors.

On comparing our results to those from historical serological prevalence studies,^11^ we observed that White and Indian South Africans share similar alleles for the RHCE*ce/ce, *Ce/cE, KEL*K_KPB_JSB (k–), FY*A, FY*A and YT*A, YT*B genotypes.^11^ Similarly, common red cell antigens among the Black and mixed race South Africans were the RHCE*cde/Cde, KEL*k_KPB_JSB/JSA and FY*A/B_GATA.^11^

According to the Department of Statistics South Africa,^10^ the four major race groups are defined as Black, White, Indian and mixed race people while internationally and outside Africa, the broadly defined ethnic groups are termed Caucasian, African American, Asian, other or mixed race. It has been proposed that global emigration patterns account for the red cell antigens of White South Africans being related to Caucasians, whereas Black and mixed race South Africans are similar to African Americans and Indian South Africans similar to Asians, and in some cases, Caucasians.^11,12,13,14^ In this study, the presence of the RHCE*Cde/cde (r’r), *Cde/cdE (r’r”), *cDe/cDe(Ro), KEL*K (k–), KEL*KPA (Kpb–) and LU*A (Lub–) in White South Africans matched the findings in Caucasian populations of Italy, Netherlands, America, Spain, Germany and Austria.^15,16,17,18,19^ Further, the rare E-Hrb- and E-HrS- found among Black South Africans matched findings in the African American population.^20,21,22^ The JS*A,JS*A genotype (Jsb–) was found in three Black South Africans and one Indian donor which is not common in South Africa. However, this phenotype is present in America and Japan and is reportedly associated with Asian populations.^12,23^ The detection of the RHCE*cDe/cDe (Ro) in all the race groups represented in the study contradicts the assumption of a race-specific blood type. Interestingly, the RHCE*cDe/cDe (Ro) was most detected in the Black South Africans but only found in the Netherlands.^16^ This finding suggests that this is a very rare blood type associated with African ancestry. The absence of the Yta(Yta–) Cartwright antigen shown by the YT*B,YT*B genotype, was found in one Indian South African but was not detected in a study by Kahar and Patel,^24^ which surveyed the prevalent red cell phenotypes in central India. The n of the Yta(Yta–) can be attributed to the small study size or area of India sampled. The Yta- antigen was also identified in Italy,^15^ America,^17^ Spain,^18^ Japan^23^ and Canada^25^ prompting a decision to expand screening for this rare antigen in South Africa to all race groups.

Similar to the European study by Finning et al.,^26^ three of the negative for HFA Js(b–), Kp(b–) and Jk(b–) were also found in the current study. Although there have been suggested similarities between South African White donors and Caucasians or Europeans, it is evident that some uniqueness exists between the two geographic regions. The Dib–, RHCE*CeRN, Dia–, Mia+, Cob+, Lua+, Dob+, which were unique to the European study by Finning,^26^ were absent in the present study possibly due to the small sample size. More studies will be required to interrogate and investigate the underlying reasons for these potential differences at a genetic level or whether this pattern changes and becomes more similar when more donors are tested.

The RHCE*CE/Ce genotype is a rare Rh subtype (R_z_R_1_) that was found in one mixed race South African. A study completed by Sharma et al.^27^ reported R_z_R_1_ to be found in 6% of Native Americans and 2.2% of Indians in Central India. The low prevalence antigen RHCE*Cw was found in two White South Africans only.

The ID CORE XT assay does not differentiate the HrB– (Rh:-34) from the hrB– (Rh:-31). The formation of anti-HrB is due to the absence of the high-frequency antigen HrB (HrB–) and anti-hrB (Rh:-31) is an ‘anti-e-like’ antibody. The absence of the ‘E’ antigen together with the HrB– (E-HrB)– indicates the true rare blood type. Besides, the ID CORE XT assay does not differentiate the HrS– (Rh:-18) from hrS– (Rh:-19) subtype. Similar to the HrB– subtype, the absence of the ‘E’ antigen combined with the HrS– subtype indicates the true rare antigen type. This study had revealed a possible differentiation of the two HrB–/hrB– and HrS–/hrS– types due to the presence of specific single nucleotide polymorphisms in the genotype and in comparison to genotype patterns produced when historically known HrB– (Rh:-34) samples are genotyped. This is reflected in Table 2 where the r’s haplotype with the presence of the 733G and 1006T pointed to the rare HrB– type while the presence of homozygous ceAR or with the single nucleotide polymorphism 712G was an indication of the HrS– rare blood type. A total of 12 HrB– and six HrS– donors were reported in this study as these two rare types originated in South Africa, hence found in more of our South African donor population.^20,21^

The rare negative high-frequency antigens k–, Kp(b–), Jk(b–), Jo(a–), Hy– and Lu(b–) were found only in White donors and this is consistent with reports in Europeans and Caucasians from America and Europe.^28^

The Fy(a-b–) rare phenotype was not identified in the current study and may be attributed to the purposive sampling techniques used in the study. The GATA mutation identified by the ID CORE XT assay predicts Fy(a–,b–); however, this is not the true rare phenotype, but rather an assay limitation.

The genotype GYPB*S, GYPB*S or s– phenotype is currently not listed as a rare type in South Africa as it is not difficult to locate; however, s– is listed on the rare donor files of Spain^18^ and Japan.^23^ Although the S-s-U– phenotype predicted by the GYPB*deletion is rare in Caucasians and found mainly in African Americans,^17^ it was found in one White and one Black donor in the present study. This suggests the presence of this rare blood type among White South Africans. Interestingly, this study revealed many null alleles among the Ss alleles of the MNS blood group system, which was missed by serology. These are important as the null alleles were mainly associated with the presence of the U-variants. Patients with U-variant phenotypes can only be given blood matched from U-variant donors or risk the formation of anti-U antibodies that will result in transfusion reactions.

Three red cell antigens, the Jk(a-b–), Co(a–) and Ko, were not identified in the present study which could be explained by the small sample size. However, donors for these three antigens were previously active rare donors on the South African Rare Donor file but became lapsed donors having not donated blood in over 3 years.^29^ This highlights the need to increase stores of rare blood units in frozen storage so that patient-donor blood matching is not challenged by the lack of active blood donors. Mass-scale cost-effective red cell genotyping can be used to screen donors routinely for this purpose in line with international practice as in the United States.^30^

The most important finding of this study was that among the mixed race group, 73% (8/11) of the donors showed the presence of rare antigens. Thus, mass-scale rare donor screening among mixed race donors can increase the pool of donors with rare blood types.

### Limitations

A limiting factor of the ID CORE XT assay is that several rare red cell antigens in South Africa such as the Rhnull, Kn(a–), Lan–, Bombay O_h_, Vel–, Adult I–, Ge–, Inb–, rare ABO subtypes and RhD/RhCE hybrids are not covered by the assay. Due to the small study sample size, genotypic findings must be confirmed in large-scale studies that include all South African ethnic groups that will be representative of the South African donor population.

The study is the first comprehensive red cell genotyping study undertaken at the South African National Blood Service and for South Africa. The extensive gene/antigen coverage of 10 blood groups systems in a single test translates to cost efficiencies, the possibility of high-throughput testing^29^ and easier detection of rare blood types present in more than one blood group. This study is also hypothesis-generating, and a few critical areas for future studies have been identified.

### Conclusion

This study has highlighted that random screening using molecular red cell genotyping increased the chances of finding rare donors by 15% (*n* = 14/92 random donors screened). It was further concluded that targeted screening using Rh subtypes associated with race will increase the likelihood of identifying more rare blood donors.

The competition between various commercial companies to provide faster, cost-effective genotyping kits with multiplex ability and high-throughput volumes has made it possible to introduce affordable mass-scale genotyping in South Africa. This will also enable the creation of a substantial local South African red cell genotype database that can be expanded to include the rest of Africa.
